# The Influence of Hyperthyroid Metabolic Status on the Coagulation and Fibrinolysis System and the Risk of Thrombosis: A Prospective Cohort Study

**DOI:** 10.3390/biomedicines13081869

**Published:** 2025-08-01

**Authors:** Manuela Andrea Hoffmann, Anne Zinndorf, Florian Rosar, Inge Scharrer, Nicolas Fischer, Tobias Gruebl, Pia-Elisabeth Baqué, Stefan Reuss, Mathias Schreckenberger

**Affiliations:** 1 Department of Nuclear Medicine, University Medical Center of the Johannes Gutenberg-University, 55131 Mainz, Germanyflorian-rosar@uks.eu (F.R.); pia-elisabeth.baque@unimedizin-mainz.de (P.-E.B.); reuss@uni-mainz.de (S.R.); mathias.schreckenberger@unimedizin-mainz.de (M.S.); 2 Institute for Preventive Medicine of the German Armed Forces, 56626 Andernach, Germany; 3 Department of Nuclear Medicine, Saarland University Medical Center, 66421 Homburg, Germany; 4 Department of Haematology of the Medical Clinic and Policlinic, University Medical Center of the Johannes Gutenberg-University, 55131 Mainz, Germany; 5 Department of Human Medicine, University of Witten/Herdecke, 58455 Witten, Germany; simplissimus@gmx.de; 6 Department of Anaesthesiology and Intensive Care, German Armed Forces Central Hospital, 56072 Koblenz, Germany; tobias.gruebl@uni-marburg.de; 7 Department of Anaesthesiology and Intensive Care, University Hospital of Gießen and Marburg, 35043 Marburg, Germany

**Keywords:** hyperthyroidism, hypercoagulability, risk of thrombosis, coagulation system, fibrinolysis system

## Abstract

**Background**: Risk assessment in hyperthyroidism remains challenging. The aim of the present study is to determine the influence of hyperthyroid metabolic status on blood clotting and an increased risk of thrombosis. **Methods**: This prospective study included 50 patients after radical thyroidectomy and ablative radioiodine therapy because of thyroid carcinoma who were compared with 50 control subjects in a euthyroid metabolic state. Latent hyperthyroid patients with basal thyroid-stimulating hormone (TSH) ≤ 0.15 mU/L on levothyroxine hormone therapy were included. The control group was selected to match the patient group based on age and sex. The evaluation data were collected using laboratory coagulation tests and patient questionnaires. A bleeding and a thrombosis score were determined. **Results**: The coagulation parameters between the patient and control groups showed statistically significant differences. In particular, the patients’ group showed a significantly shortened activated partial thromboplastin time (aPTT/*p* = 0.009) and a significantly higher plasminogen activator inhibitor 1 (PAI-1/*p* < 0.001) compared to the control group. Age, sex, and medication use were not found to influence the patients’ laboratory results. Only body mass index was higher in the patient group than in the control group. **Conclusions**: Our results support a shift in the coagulation system in latent hyperthyroid metabolism towards increased coagulability and reduced fibrinolysis. A latent hyperthyroid metabolic state appears to be associated with an increased risk of thrombosis. Further prospective cohort studies with large patient populations are needed to verify the association between (latent) hyperthyroidism and thromboembolic events as well as to determine therapeutic anticoagulation or to adjust the indication for exogenous administration of thyroid hormone.

## 1. Introduction

Pathologically altered thyroid hormones can have a decisive impact on the physiological, and metabolic processes and blood coagulation in humans [1,2,3]. Previous studies have described the association of a hypothyroid metabolic status with a tendency to bleed and hyperthyroidism with a risk of thrombosis. However, there are controversial study results that report an association with a prothrombotic state even in the case of subclinical hypothyroidism [4,5,6,7]. In addition to the risk of thrombosis in hyperthyroid patients, an association of increased cardiovascular risk and of atrial fibrillation in subclinical hyperthyroidism has been described previously [4,8,9]. The underlying mechanisms in thyroid diseases with coagulation disorders are not currently clearly understood, and risk assessment for patient outcomes remains challenging. Shih et al. suspect that the synthesis of coagulation factors is influenced by the interaction of thyroid hormones with the receptors [10]. The effect of changes in thyroid hormones on the hemostatic system can be monitored by fluctuations in the concentration of blood clotting factors. This prospective study aims to identify the influence of hyperthyroidism on the coagulation and fibrinolysis system. Our topic of interest is to evaluate the shift in latent hyperthyroid metabolism towards changes in coagulability and fibrinolysis as well as the association with thromboembolic events.

## 2. Materials and Methods

### 2.1. Patient Collective

This prospective study was carried out in the Department of Nuclear Medicine of the University Medical Center of the Johannes Gutenberg-University of Mainz, Mainz, Germany. Fifty consecutive patients with hyperthyroidism, who were treated with a radical thyroidectomy and ablative radioiodine therapy based on a thyroid carcinoma before, were included. The main inclusion criterion was a current thyroid stimulating hormone (TSH) value ≤ 0.15 mU/L (reference range at the time of the study and in the laboratory of the university: 0.4–4.9 mU/L). The study participants underwent TSH suppression therapy after radical thyroidectomy followed by Iod-131 radioablation therapy to treat existing malignant thyroid disease. Thyroid hormone therapy with levothyroxine aimed to achieve a basal TSH value of ≤0.15 mU/L. In addition to the laboratory analysis of the basal TSH value, the free thyroid values fT3 and fT4 were measured in all patients. All included patients had latent hyperthyroidism at the time of blood sampling (levels of the peripheral thyroid hormones fT3 and fT4 were within the normal range, while the TSH value was reduced).

Before the blood sample was taken, the study participants were informed in a detailed individual conversation about all study aspects of the planned examination and gave their written consent to the use of the remaining blood after the routine blood collection, which was carried out as part of conventional therapy.

All included thyroid carcinoma patients had differentiated thyroid carcinoma without known distant metastases (Table 1). Exclusion study criteria were other thyroid diseases (such as autoimmune thyroid disease, Graves’ disease, or Hashimoto’s thyroiditis) and other cancers.

### 2.2. Control Collective

As a control group, 50 study participants of the Gutenberg Health Study (GHS) were selected according to their age and sex to match the patient collective (Table 1). The GHS is a prospective, population-based, monocentric cohort study in the Rhine-Main region [11]. It was initiated by the University of Mainz, Germany as an interdisciplinary research project together with the preventive cardiology and medical clinic and polyclinic in 2007. While the first phase lasted until 2017, the second phase is expected to continue until 2027 and thus provide extensive follow-up data [11]. As an inclusion criterion, the control participants of our study had to be in a euthyroid metabolic state (TSH between 0.4 and 4.9 mU/L) at the time the blood was taken for coagulation parameters. Information about factors that could lead to a coagulation disorder was not available. The study participants had no cancer or thyroid diseases (such as autoimmune thyroid disease, Graves’ disease, or Hashimoto’s thyroiditis).

### 2.3. Laboratory Analysis and Questionnaires

Blood samples were taken as part of routine diagnostics for the thyroid cancer patients in the Department of Nuclear Medicine at the University Medical Center of Mainz, Mainz, Germany. The excess sample material was used for the laboratory coagulation tests in this study after the study participants’ explicit consent.

Before the blood samples were taken, the patient collective also filled out a questionnaire intended to obtain data regarding an increased tendency to bleeding or thrombosis.

With regard to an existing tendency to thrombosis, patients were asked about previous events associated with thrombotic disease, such as thrombosis in the past in total, thrombosis after surgery, thrombosis after bed rest, thrombosis after immobilization and after travel, thrombosis with a positive family history, spontaneous thrombosis, and family susceptibility to thrombosis. From this information a thrombosis score was determined.

Regarding an increased tendency to bleed, questions were asked about bleeding in the past in total, increased nosebleeds, gum bleeds, excess menstrual bleeding, postoperative bleeding, and bleeding tendencies in the family. Based on this data, a bleeding score was created.

In addition, the patient population was asked about their medication intake. The questionnaire considered medications that affect or may affect the hemostasis system. This included medications that are considered to be responsible for an increased tendency to thrombosis, such as corticosteroids, estrogen-containing contraceptives, immunosuppressants, tamoxifen, methotrexate, and etanercept, and medications that are responsible for a prolonged bleeding time, such as platelet aggregation inhibitors, especially acetylsalicylic acid, ibuprofen, and Marcumar.

Laboratory coagulation tests included the quick value parameter for assessing the exogenous coagulation system (also known as the thromboplastin time or TPZ) and also the coagulation factors I, II, V, VII, and X. It is used for monitoring patients taking oral anticoagulants, such as coumarin therapy. Standard values of the quick value are between 70 and 120%. The international normalized ratio (INR), a standardization of the quick value, is comparable across laboratories. It is calculated as TPZ patient plasma/TPZ normal plasma pool. The norm value is an INR close to 1.0. In our study we used the Dade^®^ INNOVIN^®^ reagent (Siemens Healthcare, Marburg, Germany).

**Figure 1:**
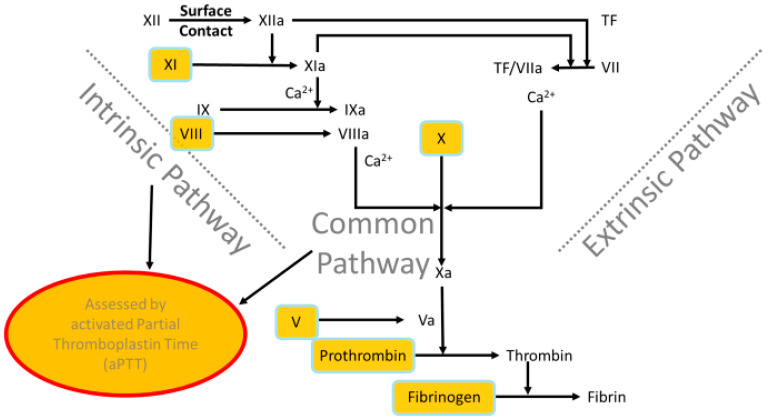
Activated partial thromboplastin time (aPTT) as a test of the intrinsic clotting pathway and the common final pathway (own illustration, adapted from M. Krapick, based on Carvalhal et al. [12]).

The determination of the activated partial thromboplastin time (aPTT) enables an assessment of the intrinsic coagulation system together with the common final pathway (Figure 1).

The laboratory value aPTT is dependent on the factors VIII, IX, XI, XII, prekallikrein, and high molecular weight kininogen. It is used to monitor heparin therapy. A prolonged aPTT occurs, for example, with a deficiency of factor VIII (hemophilia A) or IX (hemophilia B) or with heparin therapy. Whereas a shortened aPTT would indicate excessive activity of the blood coagulation system. Normal aPTT values for adults are 26–36 s according to the reference range from the central laboratory of the University Medical Center of Mainz. The Pathromtin^®^ SL reagent (Siemens Healthcare, Marburg, Germany) was used in our study.

Fibrinogen, or coagulation factor I, is proteolytically cleaved by thrombin (coagulation factor IIa) and converted into fibrin. The resulting fibrin threads and other cellular elements of the blood together form the thrombus, the end of the coagulation cascade. It is necessary for the formation of a stable clot. Decreased fibrinogen levels are found in cases of excessive coagulation, such as disseminated intravascular coagulopathy (DIC). DIC is a consumption coagulopathy due to an increased consumption of plasma coagulation factors and platelets. In addition, reduced fibrinogen synthesis may occur in liver disease. As an “acute phase protein” there is a temporary increase in levels in inflammatory diseases. Adult reference ranges are 180–350 mg/dL (according to the reference range from the central laboratory of the University Medical Center of Mainz, Mainz, Germany). There are different methods of determining fibrinogen. In our case, the principle of the so-called “derived fibrinogen” was used (HaemosIL RecombiPlasTin 2G, PT reagent; instrument: ACL TOP 750, manufacturer Instrumentation Laboratory Company, Bedford, MA, USA). This can be performed as part of a quick optical determination. As the concentration of fibrinogen increases, the intensity of the opacity increases. The amount of fibrin can therefore be determined using the extinction gradient.

D-dimers are cleavage products that are formed during fibrinolysis. The serine protease plasmin splits off dimers from under the action of factor XIII cross-linked fibrin. The D-dimer value serves as a (non-specific, as pneumonia, for example, can also be associated with an increased value) screening parameter for diagnosing thromboembolic events. The reference range is <0.49 µg/mL (according to the reference range from the central laboratory of the University Medical Center of Mainz). The determination of D-dimer concentration in our study was carried out using a particle-enhanced, immunoturbidimetric assay (HaemosIL D-Dimer HS 500; instrument: ACL TOP 750, manufacturer Instrumentation Laboratory Company, Bedford, MA, USA). Latex particles covalently loaded with monoclonal antibodies aggregate when mixed with samples containing D-dimer. The principle of immunoturbidimetry is based on the photometric measurement of the antigen-antibody reaction between the antibodies carried by polystyrene particles and the presence of D-dimer antigen.

Coagulation factor VIII, also called antihemophilic globulin A or antihemophilic factor A, together with calcium and phospholipids, is a cofactor for the coagulation factor IXa, which activates factor X. This accelerates further thrombin formation. The coagulation factor VIII is bound to its carrier protein (von Willebrand factor) in the blood and is dissolved by it under the action of thrombin. Factor VIII production is encoded by a gene on the X chromosome (Xq28). In women, low values of less than 60% indicate a possible carrier of hemophilia A, while in men it indicates varying degrees of severity of the disease. Factor VIII depends on age, blood type, inflammation, and stressful situations. The normal range is 50–150% (according to the reference range from the central laboratory of the University Medical Center of Mainz, Mainz, Germany). In our study, factor VIII-deficient plasma was used to determine the activity of factor VIII (HaemosIL Factor VIII deficient plasma, SynthASil, aPTT reagent; instrument: ACL TOP 750, manufacturer Instrumentation Laboratory Company, Bedford, MA, USA). The plasma of study participants was “spiked” with factor VIII-deficient plasma. To determine factor VIII, the sample is mixed with the “deficient plasma”, and the aPTT is then measured. If factor VIII is missing in the sample, its absence cannot be compensated for in the deficient plasma. This results in an extended PTT. The activity of factor VIII in % of the norm is determined using a reference curve that is created using dilutions of standard human plasma with the corresponding deficient plasma. The findings are reported as a percentage of the norm based on a standard curve that was created with normal plasma.

**Figure 2:**
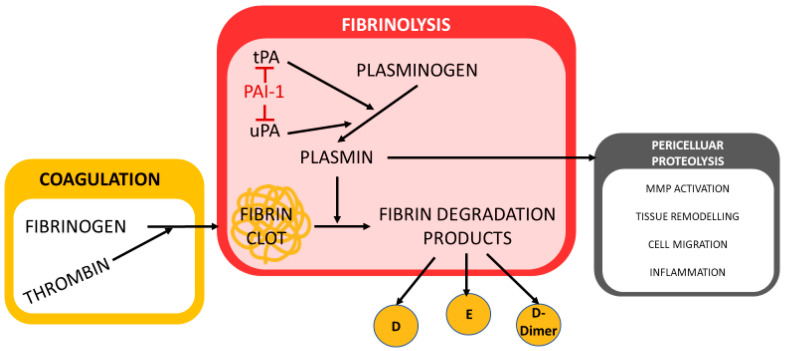
Plasminogen activator inhibitor-1 (PAI-1), an important regulator of fibrinolysis, inhibits both plasminogen activators u-PA and t-PA (own illustration, adapted from M. Krapick, based on Sillen et al. [13]).

Plasminogen is converted into the fibrinolytic enzyme plasmin by the plasminogen activators urokinase (u-PA) and tissue plasminogen activator (t-PA). As a proteolytic enzyme, plasmin has a high affinity for fibrin and is responsible for fibrin cleavage during fibrinolysis. Plasminogen activator inhibitors (PAI) are plasma proteins that act as inhibitors of fibrinolysis. Plasminogen activator inhibitor type 1 (PAI-1) (Figure 2) is the most effective PAI and inhibits both plasminogen activators u-PA and t-PA.

At elevated concentrations, PAI-1 has an antifibrinolytic effect and is clinically manifested by an increased risk for thrombosis. Reduced PAI-1 levels are often associated with hemorrhagic diathesis. The reference range is 1–7 U/mL according to the reference range from the central laboratory of the University Medical Center of Mainz. In our study, the determination of PAI-1 was carried out using the Berichrom PAI reagent from Siemens (Siemens, Marburg, Germany), which contains the enzyme urokinase. After inactivation by PAI-1 present in our plasma sample, the residual activity of the urokinase was determined via the conversion of plasminogen to plasmin. The resulting plasmin was measured photometrically by cleaving a chromogenic substrate at a wavelength of 405 nm. By creating a reference curve, the original PAI-1 activity in the sample could be determined.

### 2.4. Statistical Analysis

The statistical analyses were carried out by the statistical department of the University Medical Center of Mainz, the Institute for Medical Biometry, Epidemiology, and Computer Science (IMBEI), Mainz, Germany. For statistical analysis, The Statistical Package for the Social Sciences (SPSS) Statistics for Windows, version 22.0, was used. The normally distributed data were analyzed using Student’s *t*-test. As data were mostly non-normally distributed; or rather, the mean value of the groups showed a large variance, the non-parametric Mann–Whitney U test was used. A *p* value of <0.05 was considered statistically significant.

## 3. Results

### 3.1. Patient Characteristics

Fifty thyroid disease patients after radical thyroidectomy and ablative radioiodine therapy with TSH values ≤ 0.15 mU/L were compared with 50 control subjects in a euthyroid metabolic state at the University Medical Center of the Johannes Gutenberg-University of Mainz. To evaluate the data, the selection of the control group was matched to the patient group based on sex and age. Thirty-seven female study participants and 13 male study participants were included in both groups, with a median age of 52.0 (±13.27) years in the patient collective and of 52.9 (±10.27) in the control group.

### 3.2. Thrombosis and Bleeding Score

With regard to an existing tendency risk for thrombosis, study patients were asked about previous thrombosis, such as immobilization when confined to bed, pregnancy, and long journeys. A thrombosis score was then determined. Four patients stated that they had already suffered a thrombosis. One of these patients after a surgical procedure and with a positive family history, one patient after immobilization during a long-haul flight, one patient after being confined to bed for a long period of time, and one without known risk factors. A total of five patients reported a family history of thromboembolic events.

With regard to an increased tendency to bleed, questions were asked about increased nosebleeds, period bleeds, gum bleeds, and increased bleeding after dental treatments, surgery, and family history of bleeding tendencies. Based on this data, a bleeding score was determined. Five patients reported regular nosebleeds, ten patients had increased bleeding gums, seven patients had increased menstrual bleeding, and five patients had postoperative bleeding. Two patients reported a family history of bleeding.

At the time of blood sampling, comparison of the patients with the control study participants showed that almost the same number of patients and control subjects were taking medications that can promote the development of venous thrombosis. Six subjects and six controls took estrogen-containing medication, one patient and one participant in the control group took tamoxifen, and two patients and two controls took corticosteroids. Only one of the fifty patients (0.02%) was taking the immunosuppressants methotrexate and etanercept. The mean aPTT of the patients who took medication was slightly lower (28.42 s) than the mean aPTT of all the patients (29.74 s) (Table 2).

In the control group, the mean aPTT of the subjects who took medication was also slightly lower (31.12 s) compared to the whole control group (31.21 s) (Table 2). Patients who took medication had a lower mean value of the PAI-1 (7.53 U/mL) in comparison to all patients (11.26 U/mL). This was also the case in the control group participants (2.96 U/mL versus 4.94 U/mL) (Table 2).

In addition, we collected data of medications that can promote a tendency to bleeding taken in the patient and the control group. One patient took acetylsalicylic acid (ASA) versus seven subjects in the control group, three patients took ibuprofen versus five controls, and none of the patients took paracetamol versus two in the control group. In general, significantly fewer patients compared to controls took non-steroidal anti-inflammatory drugs (NSAIDs), but one patient took Marcumar (none in the control group). In the subject who took Marcumar, the quick value was 33% and the INR was 2.1. The mean of the mean quick value of all patients without the Marcumar case was 105.57% (with the Marcumar case: 104.02%), and the mean of the INR was 0.985 (with the Marcumar case: 1.01) (Table 2). Compared to the control group, there is no noticeable statistical difference regarding the quick value (control group: 105.68%) and INR (control group: 0.97).

### 3.3. Coagulation Parameters

In our study, there were no significant differences in the quick value between the patient and the control group (*p* = 0.372). Both values (Table 2) were close to the mean of the normal population (reference range from 70 to 120%). The average quick value of the patient group was slightly below that of the control group. There were also no significant differences between the two study populations with regard to the INR (*p* = 0.746) (Table 2). The average, INR (inversely proportional to the quick value) for the patient group was slightly higher than the control group.

**Figure 3:**
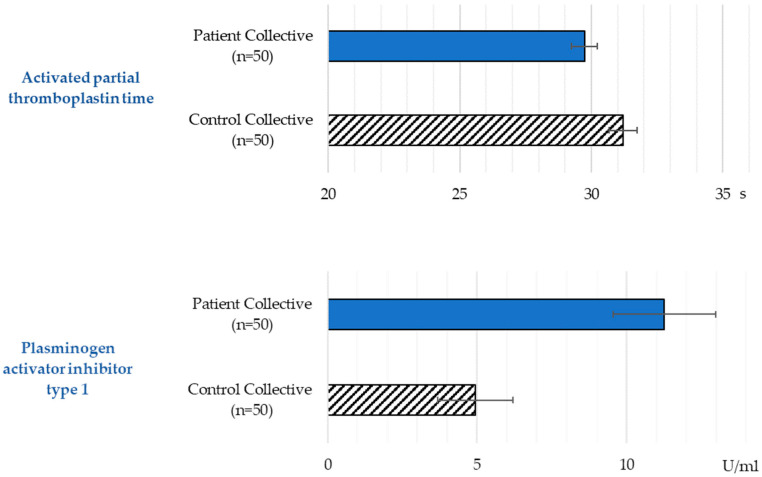
Activated partial thromboplastin time and plasminogen activator inhibitor type 1 values in the patient and the control collective (mean and standard error).

The mean values of the aPTT in both groups were within the normal range (26–36 s) (Table 2). However, the aPTT time of the patients was significantly below that of the control group (*p* = 0.009) (Figure 3).

The mean values of fibrinogen in both groups were slightly above the reference range (180 to 350 mg/dL) of the central laboratory (Table 2). The mean fibrinogen level of the control group was slightly higher than the mean value of the patient group. The Mann–Whitney U test calculated a *p*-value of 0.189.

The mean D-dimer value of the patient population was slightly above the reference range (<0.49 µg/mL) (Table 2). In comparison, the mean D-dimer increase was slightly higher in patients, but the difference was not statistically significant (*p* = 0.598).

In both groups the mean coagulation factor VIII values were within the reference range (50–150%) (Table 2). Whereas the control group mean factor VIII value (149.68%) was slightly higher than the patient population (136.67%), and the median of the patients (patients: 136.5% versus controls: 132.65%) was somewhat higher. No significant difference was found (*p* = 0.572).

The mean PAI-1 value of the patient group was clearly outside the reference range (1–7 U/mL) compared to the control group (Table 2). The Mann–Whitney U test showed a high statistical probability (*p* < 0.001).

In the patient group of our study there were fewer (8) normal weight (18.5–25 kg/m^2^) and more pre-obese (>25–30 kg/m^2^) and obese (>30 kg/m^2^) participants (40) than in the control group (18 and 32), while information regarding BMI is missing for two of the patients. There were no underweight participants in either group. The mean and median BMI in the patient group were 30.98 and 29.25 kg/m^2^ (patients) versus 27.18 and 26.37 kg/m^2^ (controls), but this data was not available in two patients.

Most aPTT values in the patient group were within the normal range regardless of BMI (32 out of 48 patients). Reduced values (<26 s) occurred equally in the group of pre-obese (2 out of 48) and obese patients (2 out of 48); none had normal weight. No pre-obese or obese patient had aPTT values above 36 s. The aPTT values in the control group were also largely within the normal range (43 out of 50 patients). A reduced value (<26 s) occurred only in one normal-weight test subject, while no pre-obese or obese controls had reduced aPTT. Four pre-obese or obese and two normal-weight controls showed higher values (>36 s).

PAI-1 values above the reference range (>7 U/mL) were found more frequently in the groups of pre-obese and obese patients (19 out of 48 patients). Reduced values (<1 U/mL) were only found in one normal-weight (18.5–25 kg/m^2^) and one obese (>30 kg/m^2^) patient, while 25 out of 48 patients were within the normal range for PAI-1 regardless of BMI.

Eight pre-obese or obese (8/50) and two normal-weighted (2/50) subjects in the control group had an elevated PAI-1 laboratory value (>7 U/mL). Twenty-one out of fifty controls showed PAI-1 values in the reference range regardless of BMI.

A total of nineteen subjects had a PAI-1 value below the reference (<1 U/mL), of which twelve were of normal weight, five were pre-obese, and two were obese.

## 4. Discussion

Abnormal thyroid hormone levels can have a decisive influence on the coagulation and fibrinolytic system and the physiological and metabolic processes in humans. Regarding the influence of thyroid hormone changes on blood coagulation, the underlying mechanisms of action are not yet clearly understood.

In this prospective study, the effect of latent hyperthyroid hormone levels on the coagulation and fibrinolysis system were investigated by measuring differences in the blood coagulation factors in patients versus “normal” control subjects. Statistically significant differences were found for the aPTT and PAI between the patient and the control groups. The aPTT provides information about the intrinsic coagulation system. A shortened time indicates hypercoagulability. PAI-1 inhibits fibrinolysis. Elevated plasma levels are associated with an increased risk of thrombosis [14]. Our results with a decreased-shortened aPTT (*p* = 0.009) and a significantly increased PAI-1 (*p* < 0.001) support the hypothesis that hyperthyroidism or latent hyperthyroidism promotes prothrombotic states. An increased quick value and a reduced INR, as parameters for assessing the exogenous coagulation system, would indicate hypercoagulability [15]. With regard to the quick value and the INR, there were no significant differences between patients and controls in our study. Fibrinogen is a substrate for both fibrin polymerization and fibrinolysis. Elevated values are found transiently in acute inflammatory diseases as part of its function as an acute phase protein. Elevated fibrinogen levels are associated with an increased risk of thrombotic and cardiovascular events. An increase in plasma fibrinogen has shown acceleration of thrombus formation in animal experimental models. Reduced values can be found due to reduced synthesis in the liver or increased conversion as part of intravascular consumption coagulopathy [16,17]. In our study, the mean values of fibrinogen in both groups were slightly above the reference range, while the mean value of the control group was slightly higher than that of the patient group (*p* = 0.189), so a conclusion regarding the risk of thrombosis is not meaningful. D-dimer is a measurement of fibrin cleavage products. As a strong biomarker of coagulation activation and secondary fibrinolysis, elevated D-dimer values are used as a diagnostic tool for venous thromboembolism. However, carcinomas or inflammatory diseases can also lead to D-dimer elevation. Therefore, the D-dimer levels are not specific for intravascular thrombus formation but can be indicative of thrombosis or embolism if certain conditions are excluded [18]. The mean value of D-dimer in our patient group is slightly above the reference range in contrast to the controls, which may support a tendency to hypercoagulability. However, our findings were not statistically significant (*p* = 0.598). Antonijevic et al. reported the role of high levels of anticardiolipin antibodies as indicators of thrombotic risk in hyperthyroidism. The elevated levels of anticardiolipin antibodies were interpreted as an epiphenomenon of immunogenic hyperthyroidism [19]. In our study, an immunogenic background of hyperthyroidism was not present.

Factor VIII deficiency is found in hemophilia A and indicates a tendency for bleeding. Elevated values of Factor VIII can be associated with deep vein thrombosis and pulmonary embolism [20]. In our study, the mean values of both groups are within the reference range, with the values of the control group being slightly higher on average than those of the patient group (149.68 versus 136.67). However, the median of the patient group is slightly higher (136.50 versus 132.65). The Mann–Whitney U test yielded a *p*-value of 0.572, indicating a random distribution of the values. Age, sex, and medication use were not found to influence the patients’ laboratory results. Only body mass index (BMI) was higher in the patient group than in the control group (mean 30.98 versus 27.18). Obesity is a risk factor for the occurrence of venous thrombosis and thus influences the coagulation system [21,22]. Almost all aPTT values in the patient group of our study were within the normal range, regardless of BMI. Reduced aPTT values occurred equally in the group of pre-obese (2) and obese patients (2). The aPTT values in the control group were also mostly in the normal range. An isolated reduced value was noted in the normal weight control group. A direct relationship between increased BMI and shortened aPTT could not be shown. According to Levine et al., obesity is associated with increased PAI-1 levels. However, small molecule inhibitors of PAI-1 have been shown to be effective in preventing nutritionally related obesity in mice. The authors reported that TM5441, an inhibitor of PAI-1, is able to reverse current obesity in mice by stimulating adipose tissue lipolysis. They suggested that the reproducibility of these positive therapeutic results in humans should be further investigated [23]. Another study by Wang et al. showed that the absence of PAI-1 can lead to increased macrophage recruitment to adipose tissue, thus promoting metabolic disorders [24]. In our patient group, elevated PAI-1 values were found, particularly in pre-obese and obese patients. The distribution of PAI-1 values was almost evenly distributed among pre-obese and obese participants (approximately 50% of PAI-1 values within the normal range, approximately 50% of PAI-1 values above the normal range). The data from the control group show a different distribution. In the group of pre-obese controls in particular, the majority of PAI-1 values (65%) were within the normal range. Only 10% of the pre-obese controls were above the normal range. In comparison, 53% of the values in pre-obese patients were elevated. In the group of obese controls, 50% of the PAI-1 values were also above the normal range. This suggests that the values are not solely related to deviating BMI distributions. However, the influence of BMI based on our results cannot be excluded. Based on the evaluation of a large Italian population cohort of 22,546 subjects, Laat-Kremers et al. emphasized the meaning of hemostasis disorders in the same regard as metabolic disorders in declaring the elevated risk of thrombosis in obese subjects [22]. Debeij et al. described already in 2014 the procoagulant effect of hyperthyroid metabolic status with elevated thyroxine (fT4) on 5000 patients through an increase in the concentration of coagulation factor VIII and von Willebrand factor [25]. Our study confirms a similar effect independent of genetic or degenerative conditions, but also in the case of subsequent metabolic conditions. It must be noted that the patients in our study had a carcinoma and had previously undergone surgery, which in and of itself is a risk for thrombosis [26,27]. In all patients, however, the carcinoma was completely removed, and the operation was more than 2 weeks previously. Nevertheless, the question of the clinical relevance of our findings still remains. In the study presented here, the patients did not have significantly elevated D-dimer values despite an increased PAI concentration and reduced aPTT. There was also no clinical diagnosis of current thromboembolic events. Thus, at least in the sample analyzed here, the procoagulatory laboratory values showed no indication of an acute thromboembolic event. However, a hyperthyroid metabolic state is becoming more established as a risk factor for thromboembolic events, which suggests that more should be directed to thyroid function in addition to the well-established risk factors such as immobilization, obesity, or procoagulant medication. Longer follow-up of such patients may indicate sustained risk of thrombosis in hyperthyroidism or the relevance of the duration of such metabolic changes. It also remains unknown to what extent the level of hyperthyroidism influences the risk of thrombosis. The strategy of anticoagulation medication could therefore depend relevantly on the metabolic status and the expected duration of this status in relation to the thyroid.

## 5. Conclusions

Our results support a relationship of an increased coagulability and a reduced fibrinolysis in the latent hyperthyroid metabolic state. Latent hyperthyroidism appears to be associated with an elevated risk of thrombosis, suggesting that more frequent coagulation monitoring in TSH-suppressed patients is justified in clinical practice. In order to more completely investigate and verify the connection and mechanism of action between (latent) hyperthyroidism and thromboembolism, to determine therapeutic anticoagulation, and to adapt the indication for exogenous thyroid hormone therapy, further prospective studies with large patient numbers are recommended.

## Figures and Tables

**Table 1:** Baseline characteristics of patient and control collective.

	Patient Collective (n = 50)	Control Collective (n = 50)
	Mean Value ± Standard Deviation	Mean Value ± Standard Deviation
Age (years)	52.0 ± 13.27	52.9 ± 10.27
Female	37	37
Male	13	13
Body mass index (kg/m^2^)	30.98	27.18
Differentiated thyroid cancer	50	0
Distant metastases (thyroid cancer)	0	0
Other cancers	0	0
Chemotherapy	0	0
Graves’ disease or Hashimotothyroiditis	0	0

**Table 2:** Coagulation parameters of the patient and control collective.

	Patient Collective (n = 50)	Control Collective (n = 50)	Reference Range *	Unit
	Mean Value ± Standard Deviation	Mean Value ± Standard Deviation		
Quick test	104.02 ± 17.7	105.68 ± 9.44	70–120	%
International normalized ratio	1.01 ± 0.21	0.97 ± 0.06	0.85–1.27	
Activated partial thromboplastin time	29.74 ± 3.77	31.21 ± 3.42	26–36	s
Fibrinogen	359.54 ± 109.1	366.21 ± 64.73	180–350	mg/dL
D-dimer	0.59 ± 0.52	0.47 ± 0.28	<0.49	µg/mL
Factor VIII	136.67 ± 29.43	149.68 ± 52.62	50–150	%
Plasminogen activator inhibitor-1	11.26 ± 12.14	4.94 ± 8.95	1–7	U/mL

* Reference range of the central laboratory at the University Hospital Mainz.

## Data Availability

The data sets for this study can be obtained upon reasonable request from the Department of Nuclear Medicine at the Mainz University Medical Center in Germany.
